# Examinations of maternal uniparental disomy and epimutations for chromosomes 6, 14, 16 and 20 in Silver-Russell syndrome-like phenotypes

**DOI:** 10.1186/s12881-016-0280-8

**Published:** 2016-03-11

**Authors:** Jana Sachwitz, Getrud Strobl-Wildemann, György Fekete, Laima Ambrozaitytė, Vaidutis Kučinskas, Lukas Soellner, Matthias Begemann, Thomas Eggermann

**Affiliations:** Institute of Human Genetics, RWTH Aachen, Pauwelsstr. 30, Aachen, Germany; Praxis für Humangenetik, Ulm, Germany; II. Department of Pediatrics, Semmelweis University, Budapest, Hungary; Department of Human and Medical Genetics, Faculty of Medicine, Vilnius University, Vilnius, Lithuania

**Keywords:** Uniparental disomy, Temple syndrome, Silver-Russell syndrome, upd(6)mat, upd(16)mat, upd(20)mat, Genomic imprinting

## Abstract

**Background:**

Silver-Russell syndrome (SRS) is a growth retardation disorder with a very broad molecular and clinical spectrum. Whereas the association of SRS with imprinting disturbances of chromosomes 11p15.5 and 7 is generally accepted, there are controversial discussions on the involvement of other molecular changes. The recent reports on the occurrence of maternal uniparental disomies of chromosomes 6, 16 and 20 (upd(6, 16, 20)mat), as well as 14q32 imprint alterations in patients with SRS phenotypes raise the question on the involvement of these mutations in the etiology of SRS.

**Methods:**

A cohort of 54 growth retarded patients with SRS features was screened for aberrant methylation patterns of chromsomes 6, 14, 16 and 20.

**Results:**

One carrier of a 14q32 epimutation was identified whereas epimutations and maternal UPD for chromosomes 6, 16 and 20 were excluded.

**Conclusions:**

Our data and those from the literature confirm that 14q32 disturbances significantly contribute to the mutation spectrum in this cohort. Furthermore, maternal uniparental disomy of chromosomes 6, 16 and 20 can be observed, but are rare. In case they occur they can be regarded as causative for clinical features.

## Background

Imprinting disorders (IDs) are a group of clinically heterogeneous congenital diseases characterized by the same types of molecular alterations (for review: [1]). The underlying molecular changes affect imprinted chromosomal regions and genes, i.e. genes that are expressed in a parent-of-origin specific manner. Nearly all currently known IDs have been identified by uniparental disomy (UPD), i.e. by the inheritance of both chromosomal homologues from the same parent. Meanwhile, both maternal and paternal UPDs have been described for nearly all human chromosomes [2]. Two types of UPD can be discriminated: uniparental heterodisomy (UPhD) where the two different alleles of the same parent are transmitted, and uniparental isodisomy (UPiD) where two identical copies of one allele of the contributing parent are present. The clinical outcome of UPD is directly related to the genetic content and size of the affected chromosomal region. If the UPD affects a chromosome harboring imprinted genes, it can result in an ID (i.e. upd(6)pat, upd(7)mat, upd(11), upd(14), upd(15), upd(20)). Furthermore, there is the potential risk of homozygosity of an autosomal-recessive mutation in case of UPiD. A third pathoetiological mechanism can be delineated from UPD formation mechanisms: many UPhDs are the result of a trisomic rescue, here the supernumerary chromosome of an originally trisomic zygote is lost, a mechanism which has been confirmed in-vivo (e.g. [3]). However, dependent on the time of the trisomic rescue and the survival probability of the trisomic cell line, aberrant cell lines might survive and this mosaic constitution might influence the phenotype.

Several UPDs do not result in specific clinical syndromes, as the affected chromosomes or chromosomal regions do not harbor imprinted genes (for review: [4]). Finally, there remains a small number of chromosomes which have either not yet been reported as UPD (e.g. upd(18)mat, upd(19)), or for which ambiguous results exist (upd(6)mat, upd(16)mat).

UPD is only one molecular occurring in IDs. In this group of congenital disorders the disturbed balance of imprinted genes expression might also be caused by deletions or duplications by point mutations or aberrant methylation (epimutations) affecting the regulative regions and genes.

Furthermore, there is a molecular (and clinical) overlap between the different entities. Thus, IDs are molecularly and clinically heterogeneous, making their diagnosis difficult.

An ID with a very broad molecular and clinical spectrum is Silver-Russell syndrome (SRS, OMIM 180860), a growth retardation syndrome associated with a characteristic face, relative macrocephaly and asymmetry (for review: [5]). Whereas the association of SRS with disturbances of chromosomes 11p15.5 and 7 is widely accepted, there are controversial discussions on the involvement of other molecular changes. For instance, comprehensive clinical studies in the recently described Temple syndrome (TS14, OMIM 616222) and molecular analyses of SRS cohorts have shown that chromosome 14q32 alterations contribute to the molecular spectrum in SRS [5–7]. Furthermore, the recent reports on the occurrence of upd(6)mat, upd(16)mat and upd(20)mat in patients with SRS phenotypes raises the question on the involvement of these mutations in the etiology of disease [5, 8, 9].

In the following we report on the analysis for these variants in a cohort of growth retarded patients with SRS features and discuss our results in context with the literature.

### Study population

The study cohort consisted of 54 patients with intrauterine and postnatal growth retardation referred as SRS for routine diagnostic testing. Clinical scoring for SRS according to the modified classification system suggested by Bartholdi et al. [10, 11] and the recently published Netchine-Harbison-Score [5] revealed that 39 %/43 % of patients showed clinical signs corresponding to SRS, whereas 61 %/57 % of patients exhibited only single features reminiscent for SRS in addition to growth retardation. The latter group was analyzed for UPD as the clinical picture of patients with one of the rare UPDs may differ from the typical SRS criteria. The study was approved by the Ethical committee of the University Hospital, RWTH Aachen, Germany (EK159/08).

## Methods

Molecular changes of the imprinting control regions in 11p15.5 were excluded by methylation-specific multiplex ligation probe-dependent amplification (MS-MLPA) using a commercially available assay (ME030 BWS/SRS version C3; MRC Holland Amsterdam/The Netherlands). Point mutations in *CDKN1C* and *IGF2* had been excluded before. Upd(7)mat was excluded by MS single nucleotide primer extension assays (MS-SNuPE) [12] or MS-MLA (ME032-A1). The same analyses allowed the analysis for aberrations of the DMRs at the imprinted loci *PLAGL1* in 6q24, *IGF2R* in 6q25, and *MEG3* in 14q32. Screening for upd(20)mat was carried out by another MS-MLPA assay (ME031 GNAS, version B1).

For detection of upd(16)mat microsatellite typing was performed if at least one parental DNA sample was available (*n* = 30), using markers spanning chromosome 16 (D16S513, D16S3144, D16S3069, D16S2062, D16S500, D16S403).

For the other cases (*n* = 24) a specific MS-SNuPE assay was developed. MS-SNuPE is a technique to analyze the methylation status of individual CpG sites [12, 13]. After sodium bisulfite treatment converting unmethylated cytosine into uracil, the target regions of *ZNF597* maternal and paternal DMR were amplified by using polymerase chain-reaction (PCR). For MS-SNuPE analysis oligonucleotides that hybridize directly besides a CpG of interest inside those DMRs are used for primer elongation reaction. For single nucleotide sequencing only fluorescent marked ddNTPs are used, resulting in chain termination after integration of only a single nucleotide base. In case of methylated cytosine in the template DNA cytosine is inserted, in case of unmethylated cytosine thymidine. Comparison of both alleles allow a quantification of the methylation state in comparison to controls. Detection of the fluorescent signals and data analysis are carried out by AB3130 Genetic Analyzer and GeneMapper Software (Applied Biosystems, Darmstadt/Germany). The assay was designed to analyse both a maternal and paternal differentially methylated region (DMR) in *ZNF597* 3’ DMR locus of chromosome 16 (maternal DMR: hg19:chr16:3,481,801-3,482,332; paternal DMR: hg19:chr16:3,493,061-3,493,648). For both regions three different MS-SNuPE primers were designed to analyze the methylation status of the targeted CpG. Primer sequences and assay conditions are available on request.

## Results

By analysis of a cohort of 54 growth-retarded patients referred for genetic testing of SRS without 11p15.5 defects and upd(7)mat, screening for aberrant methylation patterns at the imprinted loci in 6q24 (*PLAGL1*) and 6q25 (*IGF2R*) by MS-SNuPE did not reveal any abnormality. Upd(20)mat, epimutations or copy number variations at the imprinted *GNAS* loci in 20q13.32 were also excluded by MS-MLPA in all cases.

**Fig. 1:**
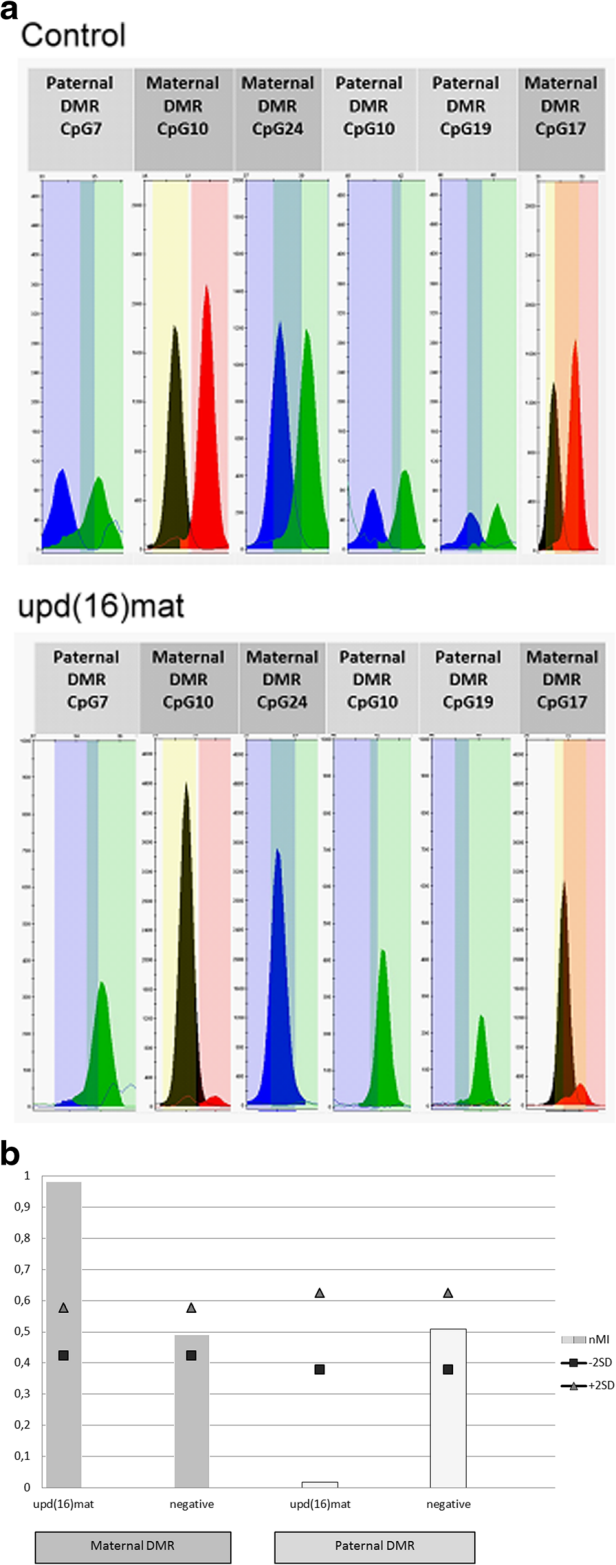
MS-SNuPE assay to identify upd(16)mat and aberrant methylation patterns at the imprinted *ZNF597* locus. **a** Raw data of the MS-SNuPE assay as displayed in the GeneMapper Software (Applied Biosystems) after the analysis with the panels for *ZNF597* from a case with upd(16) and a normal control. The methylated alleles are represented by blue or black peaks, while the unmethylated alleles are displayed in green or red. **b** Methylation index (MI) plotted on the ordinate; square and the triangle indicate the −/+ 2 standard deviation range of MI identified in a control group with normal methylation pattern. In the maternal DMR is shown in dark grey: In case of upd(16) a hypermethylation (MI = 0.98) of the maternal DMR is present, whereas the negative control has a MI around 0.5. The paternal DMR (light grey) exhibit a hypomethylation (MI = 0.02) in upd(16)mat and normal patterns of methylation in a negative control

The MS-SNuPE based test for upd(16)mat analysis was validated by analyzing two upd(16)mat carriers from our own cohort (Fig. 1), the methylation range was determined by testing 14 control DNA samples For each CpG an individual two standard deviation was calculated and used for further analysis. By application of this assay we could exclude aberrant methylation at the *ZNF597* locus in 16p13.3 in 24 patients, in additional 30 families upd(16)mat was excluded by STR typing.

Whereas screening for UPDs of chromosomes 6, 16 and 20 was negative in our cohort, we identified one patient with a hypomethylation of the MEG3 and IG-DMR in 14q32 by MS-MLPA (Table 1). Upd(14)mat and deletion of 14q32 was excluded by microsatellite typing and the MS-MLPA copy number run.

**Table 1:** Overview on the number of carriers of upd(6)mat, upd(16)mat, TS14 and upd(20)mat patients from the literature and clinical data on our patients with 14q32 epimutation

Congenital ID	upd(6)mat	SRS		TS14	upd(16)mat^a^	upd(20)mat	Patient with 14q32 hypomethylation
Reference	available on request	[24]		[7]	available on request	[9]	
Number of patients	13	20	44	51	72	15	
Cases with chromosomal disturbances	4/9				40/44	3/12	
Cases with normal phenotype	1				<10^c^		
Major Clinical and Overlapping Findings							
IUGR (<P10)	53.8 % (7/13)	70 %	82 %	87 %	74 % (53/72)	100 %	yes
PNGR (<P10)	3^b^cases	65 %	57 %	79 %	1 case	100 %	yes
Asymmetry	1 case	30 %	68 %	4 %			no
Relative macrocephaly	1 case	90 %	70 %	56 %		1 case	no
Relative macrocephaly	1 case				1 case		no
Hypotonia	1 case	45 % (*n* = 143) [25]		93 %		1 case	yes
Abdominal wall defects	1 case	rare			1 case		no
Glycemic disorder		hypoglycemia: 24 %	hypoglycemia: 19 %; diabetes type 2 reported in later life	hypoglycemia, diabetes type 2 reported in later life	hypoglycemia: 1 case		yes
Precocious puberty		frequent	frequent	86 %			too young
Mental retardation		global delay: 65 %	global delay: 20 %	39 %	1 case		
Speech delay		50 %	39 %				yes
Motor delay	2 cases	50 % (7/14)	76 % (26/34)				yes
Behaviour		20 %	9 %				
Feeding difficulties	1 case	90 %	84 %	43 %		7 cases	yes
Seizures	1 case				1 case	1 case	
Excessive Sweating		75 %	64 %				
Scoliosis		5 %	9 %	23 %		1 case	
Adipostas			reported in later life [26]	yes			
Dysmorphic/typical facial gestalt	1 case	triangular face			6 cases	mild	yes
Dlinodactyly/finger abnormalities		45 %	75 %			5 cases	yes
Ear abnormalities		low set posterior	low set posterior				
Otitis media		20 %	14 %	17.6 % (9/51)			

^a^the majority of patients was identified prenatally, 13 ended as therapeutic abortions. Data on postnatal development are scarcely available. ^b^among them a patient with *CUL7* mutation – 3 M syndrome; ^c^[23]

The girl was born to an unrelated and healthy German couple. The pregnancy was achieved by assisted reproduction technology (ART) because of azoospermia of the father with proven compound heterozygosity for p.F508del and p.R117H in the *CFTR* gene. Two previous pregnancies of the parents arising after intracytoplasmatic sperm injection (ICSI) ended early.

At 30 gw intrauterine growth retardation (IUGR) was noted. The girl was born at 32 + 6 gestation weeks (gw) by caesarean section because of pathological CTG and decreasing movements of the child. Weight at birth was 1315 g (−1.63SD), length 41 cm (−0.97SD), and occipital frontal circumference (OFC) 29 cm (−0.89SD). Apgar scores were 9/10/10. Placenta was small, calcifications were not observed. In the first days brachycardia, apnoea and hypotonia were reported. Feeding difficulties required gastric feeding.

**Fig. 2:**
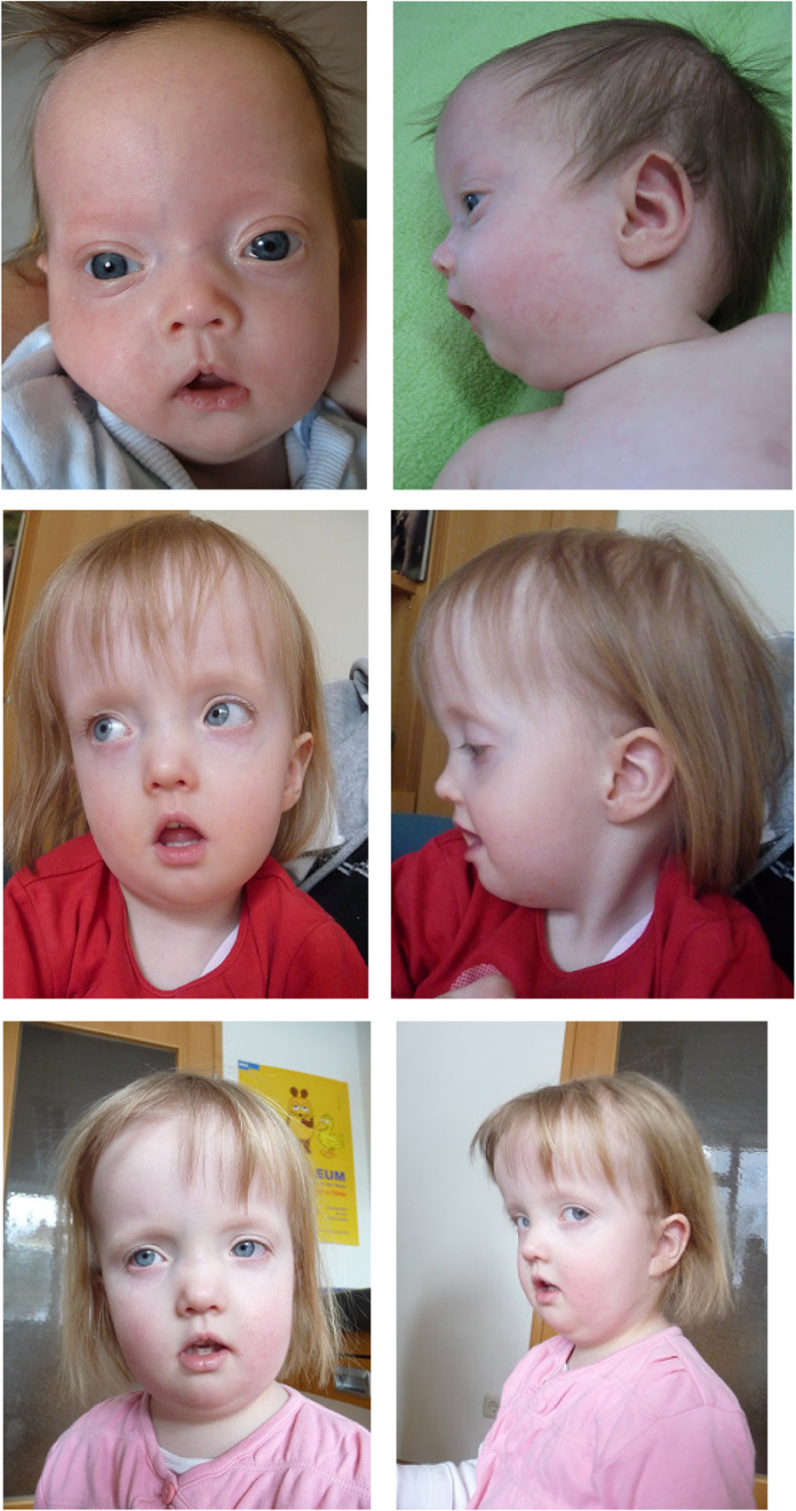
Clinical pictures of the patient with 14q32 epimutation at the age of 3 months, 4 years, and 5 years (from top to bottom). Note the prominent forehead, the flat nose, the thin upper lip, the hypotonic mouth and the retrognathia. The written consent to use these photos has been signed by the legal representatives of the patients

At the age of 3 months, postnatal growth retardation persisted (53 cm (−3.21 SD), 3750 g (BMI: 13.3), OFC 36.5 cm (−3.09 SD)), and the following dysmorphisms features were documented: low set ears, a prominent forehead with a slightly triangular face, small mouth with downturned corners, and retrognathia. Hand and feet were small, clinodactyly V and syndactyly of the toes 2–4 were present. The patient suffered from recurrent hypoglycemia. Growth retardation was also reported at the age of 26 months (81 cm (−2.27 SD), 8.6 kg (BMI: 13.11), OFC 47.9 (−0.31SD)). Clinical scoring with the Netchine-Harbison indicated that she does not fit the typical SRS phenotype score (3/6 criteria)(Fig. 2).

## Discussion

The major molecular alterations of chromosomes 7 and 11 account for more than 60 % of SRS patients, thereby leaving a large diagnostic gap. Based on single reports on disturbed imprinting marks at loci on other chromosomes in growth retarded patients with SRS features (i.e. chromosomes 6, 14, 16 and 20), the contribution of these chromosomes to ID phenotypes is currently under discussion.

In the last two years it has turned out that some patients with a characteristic SRS phenotype exhibit chromosome 14q32 alterations which are usually associated with TS14. Like SRS, TS14 is typically characterised by prenatal and postnatal growth retardation, muscular hypotonia, and feeding difficulties in early childhood. Furthermore, some patients exhibit dysmorphic features characteristic of SRS [7], and this overlap leads to the identification of the 14q32 changes in patients referred for SRS testing [5, 6, 14]. With the identification of a patient with a 14q32 epimutation in our cohort we could confirm this observation. In different screening studies for TS14-associated 14q32 alterations in growth retarded patients with SRS features the detection rate ranges between 1.5 % (*n* = 1/65; [5]) and 2.4 % (*n* = 2/85; [6] in patients with typical SRS phenotype) and 1.8 % (*n* = 1/55; this study) and 0.5 % (*n* = 3/383; [14]). Thus, 14q32 testing should be considered in the diagnostic workup of patients with SRS features. Interestingly, the patient presented here was born after ICSI treatment and this is the second case with a14q32 epimutation born after ART [6]. It fits with the observation that ART procedures affect methylation status of DMRs (for review: [15]), and an increased incidence of patients with imprinting disorders among children born after ART has been reported.

The second imprinting disorder overlapping with SRS is the recently defined upd(20)mat syndrome [9]. The upd(20)mat phenotype comprises IUGR and PNGR, and severe feeding difficulties with failure to thrive, but characteristic dysmophisms are not present, illustrated by patient 6 of Mulchandani et al. [9]. This patient belongs to a cohort of 18 patients with isolated IUGR and PNGR we screened for upd(20)mat (unpublished data). Nevertheless, upd(20)mat has also been identified in an SRS patient [5], but in general it rather seems to be associated with an unspecific growth retardation phenotype. The summary of screening studies for upd(20)mat and aberrant methylation at the chromosome 20-encoded imprinted locus GNAS shows that the condition can occur in cohorts with isolated growth retardation or SRS and SRS-like phenotypes ([5, 16, 17], present data). In total, more than 270 patients with a phenotypic spectrum ranging from isolated IUGR/PNGR to SRS have been screened, leading to the identification of three patients carrying a upd(20)mat [5, 9, 17]. Thus upd(20)mat testing might be considered in the diagnostic workup of growth retardation after exclusion of clinically more obvious molecular disturbances. Up to now, UPD is the only type of molecular change identified in upd(20)mat patients, but comparable to other imprinting disorders other types of molecular changes can be expected.

In contrast to TS14 and upd(20)mat, the clinical consequences of upd(6)mat and upd(16)mat are ambiguous, as both constitutions have been detected in individuals with clinical features, i.e. IUGR, as well as in healthy carriers.

Among the 13 carriers of upd(6)mat reported so far (Table 1), 12 showed clinical features. IUGR was reported in seven of them, postnatal growth retardation in three patients, among them one with homozygosity of a *CUL7* mutation [18]. *CUL7* mutations are associated with 3 M syndrome, a differential diagnosis of SRS. In three cases, pathogenic mutations in already known disease genes could be identified. Another upd(6)mat has been detected in a SRS patient who also carried a 11p15 duplication due to a familial translocation [8]. However, one healthy carrier of upd(6)mat has been reported [19], thus it is questionable whether this constitution is a separate entity as suggested by [20] or whether the clinical features in the carriers reported so far are attributable to (undetected) chromosomal aberrations.

Screening of patients with growth retardation phenotypes does not reveal a major significance of this alteration, in a total of more than 380 growth retardation referred as SRS for routine diagnostic testing [14] as well as in the present cohort it was not observed. Thus, upd(6)mat might be detected coincidentally, but does not contribute to a specific phenotype. However, in case it is detected in clinically striking carriers, it is convincing that the upd(6)mat contributes to the features.

The situation is similar for upd(16)mat though this aberration has been reported more frequently (Table 1). The frequency of upd(16)mat is not surprising as it is the consequence of trisomy rescue and trisomy 16 is the most common autosomal trisomy in human abortions. Trisomy 16 itself is lethal for the fetus, but in case of trisomy rescue it is compatible with life. As a consequence of this formation mechanism, several of the reported upd(16)mat cases are associated with trisomy 16 mosaicism in the placenta. The upd(16)mat has been suspected to have clinical consequences though healthy carriers have been reported (for review: [4]). However, the heterogeneity of the birth defects suggests that the phenotype might rather be influenced by placenta insufficiency or (undetected) mosaicism for trisomy 16 than by the UPD itself [21]. The possibility that upd(16)mat is associated with imprinting is difficult to assess due to the trisomy 16 mosaicism present in many cases. However, by an extensive clinical analysis of a series of mosaic trisomy 16 cases (*n* = 83) including upd(16)mat (*n* = 33), Yong and coworkers [22] convincingly concluded that upd(16)mat might be associated with more severe growth retardation in-utero and an elevated risk of malformation. This hypothesis is compatible with the recent description of a upd(16)mat carrier exhibiting a typical SRS phenotype [5].

## Conclusion

The results of screening for disturbed imprinting of chromosomes 6q24, 14q32, 16 and 20q13 confirm that the 14q32-associated disturbances significantly contribute to the mutation spectrum in SRS and related growth retardation phenotypes. Furthermore, maternal uniparental disomy of chromosomes 6, 16 and 20 can be observed in this group of patients, but they are rare. While it becomes obvious that upd(20)mat is a new imprinting syndrome, the association between upd(6)mat and upd(16)mat and specific clinical features is unclear. At least for upd(16)mat it has been suggested that imprinted genes contribute to the etiology of IUGR. Thus, in the rare cases they occur both UPDs can be regarded as causative for clinical features.

In summary, testing for the imprinted loci in 14q32 should be considered in the diagnostic workup of growth retardation phenotypes, whereas the implementation of testing of the other loci requires further studies to estimate their significance. Furthermore, the patient with epimutation in 14q32 shows that UPD is not the only type of molecular change, as already known for the other imprinting disorders. Thus microsatellite analysis restricted to the identification of UPD is not sufficient but needs to be replaced by methylation-specific assays, e.g. MS-MLPA. The future application of tests aiming on the simultaneous detection of aberrant imprinting at multiple loci (“multilocus testing”) will provide the tool to detect all aberrations in parallel, and will furthermore allow the detection of apparently rare epigenetic constitutions.

## Abbreviations

ART: assisted reproduction technology
DMR: differentially methylated region
ICSI: intracytoplasmatic sperm injection
IUGR: intrauterine growth retardation
PNGR: postnatal growth retardation
SRS: Silver-Russell syndrome
TS14: temple syndrome
UPD/UPhD/UPiD: uniparental (hetero/iso)Disomy

## References

[1] Eggermann T, Netchine I, Temple IK, Tumer Z, Monk D, Mackay D, Gronskov K, Riccio A, Linglart A, Maher ER (2015). Congenital imprinting disorders: EUCID.net - a network to decipher their aetiology and to improve the diagnostic and clinical care. Clinical epigenetics.

[2] Liehr T. Cases with uniparental disomy [http://upd-tl.com/upd.html]. Accessed Nov 2015.

[3] Petit F, Holder-Espinasse M, Duban-Bedu B, Bouquillon S, Boute-Benejean O, Bazin A, et al. Trisomy 7 mosaicism prenatally misdiagnosed and maternal uniparental disomy in a child with pigmentary mosaicism and Russell- Silver syndrome. Clin Genet. 2012;81(3):265–71. 10.1111/j.1399-0004.2010.01621.x 21204802

[4] Kotzot D, Utermann G (2005). Uniparental disomy (UPD) other than 15: phenotypes and bibliography updated. Am J Med Genet A.

[5] Azzi S, Salem J, Thibaud N, Chantot-Bastaraud S, Lieber E, Netchine I, Harbison MD (2015). A prospective study validating a clinical scoring system and demonstrating phenotypical-genotypical correlations in Silver-Russell syndrome. J Med Genet.

[6] Kagami M, Mizuno S, Matsubara K, Nakabayashi K, Sano S, Fuke T, Fukami M, Ogata T. Epimutations of the IG-DMR and the MEG3-DMR at the 14q32.2 imprinted region in two patients with Silver-Russell syndrome-compatible phenotype. Eur J Hum Genet. 2015;23(8):1062-7. 10.1038/ejhg.2014.234 PMC4795120 25351781

[7] Ioannides Y, Lokulo-Sodipe K, Mackay DJ, Davies JH, Temple IK (2014). Temple syndrome: improving the recognition of an underdiagnosed chromosome 14 imprinting disorder: an analysis of 51 published cases. J Med Genet.

[8] Begemann M, Spengler S, Gogiel M, Grasshoff U, Bonin M, Betz RC, Dufke A, Spier I, Eggermann T (2012). Clinical significance of copy number variations in the 11p15.5 imprinting control regions: new cases and review of the literature. J Med Genet.

[9] Mulchandani S, Bhoj EJ, Luo M, Powell-Hamilton N, Jenny K, Gripp KW, Elbracht M, Eggermann T, Turner CL, Temple IK et al. Maternal uniparental disomy of chromosome 20: a novel imprinting disorder of growth failure. Genet Med. 2015. 10.1038/gim.2015.103 26248010

[10] Bartholdi D, Krajewska-Walasek M, Ounap K, Gaspar H, Chrzanowska KH, Ilyana H, Kayserili H, Lurie IW, Schinzel A, Baumer A (2009). Epigenetic mutations of the imprinted IGF2-H19 domain in Silver-Russell syndrome (SRS): results from a large cohort of patients with SRS and SRS-like phenotypes. J Med Genet.

[11] Spengler S, Begemann M, Ortiz Bruchle N, Baudis M, Denecke B, Kroisel PM, Oehl-Jaschkowitz B, Schulze B, Raabe-Meyer G, Spaich C (2012). Molecular karyotyping as a relevant diagnostic tool in children with growth retardation with Silver-Russell features. J Pediatr.

[12] Begemann M, Leisten I, Soellner L, Zerres K, Eggermann T, Spengler S (2012). Use of multilocus methylation-specific single nucleotide primer extension (MS-SNuPE) technology in diagnostic testing for human imprinted loci. Epigenetics.

[13] Gonzalgo ML, Liang G (2007). Methylation-sensitive single-nucleotide primer extension (Ms-SNuPE) for quantitative measurement of DNA methylation. Nat Protoc.

[14] Eggermann T, Heilsberg AK, Bens S, Siebert R, Beygo J, Buiting K, Begemann M, Soellner L (2014). Additional molecular findings in 11p15-associated imprinting disorders: an urgent need for multi-locus testing. J Mol Med (Berl).

[15] Uyar A, Seli E (2014). The impact of assisted reproductive technologies on genomic imprinting and imprinting disorders. Curr Opin Obstet Gynecol.

[16] Turner CLS, Mackay DM, Callaway JLA, Docherty LE, Poole RL, Bullman H, Lever M, Castle BM, Kivuva EC, Turnpenny PD (2010). Methylation analysis of 79 patients with growth restriction reveals novel patterns of methylation change at imprinted loci. Eur J Hum Genet.

[17] Eggermann T, Mergenthaler S, Eggermann K, Albers A, Linnemann K, Fusch C, Ranke MB, Wollmann HA (2001). Identification of interstitial maternal uniparental disomy (UPD) (14) and complete maternal UPD(20) in a cohort of growth retarded patients. J Med Genet.

[18] Sasaki K, Okamoto N, Kosaki K, Yorifuji T, Shimokawa O, Mishima H, Yoshiura KI, Harada N (2011). Maternal uniparental isodisomy and heterodisomy on chromosome 6 encompassing a CUL7 gene mutation causing 3 M syndrome. Clin Genet.

[19] Hong M, Beischel L, Rakeach J, Wallace D, Knops J, O’Connor K, Johnson J, Ibrahim J. Prenatal detection of maternal uniparental disomy (UPD) of chromosome 6 and’rescue’ of trisomy 6. 57th annual meeting of the ASHG, Abstract No. 2048. 57. San Diego: Am Soc Hum Genet; 2007.

[20] Poke G, Doody M, Prado J, Gattas M (2013). Segmental maternal UPD6 with prenatal growth restriction. Mol Syndromol.

[21] Ledbetter DH, Engel E. Uniparental disomy in humans: development of an imprinting map and its implications for prenatal diagnosis. Hum Mol Genet 1995, 4 Spec No:1757–1764. 10.1093/hmg/4.suppl_1.1757 8541876

[22] Yong PJ, Marion SA, Barrett IJ, Kalousek DK, Robinson WP (2002). Evidence for imprinting on chromosome 16: the effect of uniparental disomy on the outcome of mosaic trisomy 16 pregnancies. Am J Med Genet.

[23] Takenouchi T, Awazu M, Eggermann T, Kosaki K: Adult Phenotype of Russell-Silver Syndrome: A Molecular Support for Barker-Brenner’s Theory. Congenit Anom 2015. doi:10.1111/cga.12105. [Epub ahead of print]. 10.1111/cga.12105 25639378

[24] Wakeling EL, Amero SA, Alders M, Bliek J, Forsythe E, Kumar S et al. Epigenotype-phenotype correlations in Silver-Russell syndrome. J Med Genet. 2010; 47:760-8. 10.1136/jmg.2010.079111 PMC2976034 20685669

